# A conformational change of the domain IV S6 segment of the voltage-gated sodium channel during inactivation

**DOI:** 10.1186/2050-6511-13-S1-A60

**Published:** 2012-09-17

**Authors:** Vaibhavkumar S Gawali, René Cervenka, Péter Lukács, Xaver Koenig, Lena Rubi, Ágnes K Mike, Karlheinz Hiber, Hannes Todt

**Affiliations:** 1 Department of Neurophysiology and Neuropharmacology, Center for Physiology and Pharmacology, Medical University of Vienna, 1090 Vienna, Austria

## Background

In voltage-gated Na^+^ channels the S6 transmembrane segment of domain IV (DIV-S6) is part of the lining of the inner part of the pore. It is of pivotal importance for inactivation gating. We recently showed that amino acid I1581 of DIV-S6 (rNa_V_1.4 amino acid numbering) is extraordinarily sensitive to both local and distal mutations suggesting a unique role in coupling of voltage-sensor movements to conformational changes in the pore. To date the only structural information relevant to voltage-gated Na^+^ channels can be derived from the recently crystallized bacterial channel Na_V_Ab. In this structure the amino acid homologous to I1581 faces the lipid phase and is in close spacial relationship to the voltage-sensing apparatus. If this arrangement holds true for the eukaryotic Na^+^ channel then site 1581 should not be exposed to bulk solution.

## Methods

The following methods were used: site-directed mutagenesis of amino acids in the S6 segment of domain IV of the rNa_V_1.4 channel; heterologous expression of the constructs in tsA 201 cells and *Xenopus laevis* oocytes; exploration of the kinetic effects of the mutations and gating sensitivity of the amino acid residue in the S6 segment of domain IV of the rNa_V_1.4 channel by whole-cell patch clamp and two-electrode voltage clamp technique.

## Results

We tested the hypothesis by replacing I1581 by a titrable histidine. In wild-type channels changing the pH of the bulk solution from 7.4 to 8.2 had no effect on the voltage-dependence of fast inactivation. However, in I1581H the same change in pH resulted in a 9.51 ± 1.98 mV hyperpolarizing shift (p < 0.05) of the voltage-dependence of fast inactivation.

## Conclusions

The data suggest that during inactivation site 1581 is at least partially exposed to the bulk solution and not completely embedded in the lipid phase. The DIV-S6 segment may undergo a conformational change during inactivation, most likely a rotational movement, which allows access of external protons to site 1581.

